# Evidence of fibrinogen as a target of citrullination in IgM rheumatoid factor-positive polyarticular juvenile idiopathic arthritis

**DOI:** 10.1186/1546-0096-10-S1-A119

**Published:** 2012-07-13

**Authors:** Brooke E Gilliam, Melinda R Reed, Anil K Chauhan, Amanda Dehlendorf, Peri H Pepmueller, Terry L Moore

**Affiliations:** 1 St Louis University HSC, St Louis, MO, USA

## Purpose

The role of anti-cyclic citrullinated peptide (anti-CCP) antibodies in juvenile idiopathic arthritis (JIA) has become better understood; however, the identity of the target proteins of this modification remains elusive. We evaluated serum from patients with various subtypes of JIA to investigate the presence of anti-citrullinated fibrinogen and anti-citrullinated α-enolase antibodies, and their association with rheumatoid factor (RF) and anti-CCP antibodies.

## Methods

Sera were obtained from 96 JIA patients, 19 systemic lupus erythematosus (SLE) patients, and 10 healthy children. All sera were measured for antibodies against citrullinated and native fibrinogen and α-enolase by enzyme-linked immunosorbent assay. All results were correlated with clinical and laboratory parameters.

## Results

Thirty-one (32%) JIA patients demonstrated reactivity to citrullinated fibrinogen and 9 (9%) to citrullinated α-enolase. Reactivity to citrullinated fibrinogen and α-enolase was predominantly found in IgM RF-positive polyarthritis patients (81% and 18%, respectively). Anti-citrullinated fibrinogen antibodies were significantly elevated in JIA patients when compared to the healthy and SLE control groups (p<0.05). Antibody reactivity patterns showed that the largest group of JIA patients reacted only with fibrinogen (17%). Ninety-three percent of JIA patients positive for IgG anti-CCP antibodies also reacted with citrullinated fibrinogen, making up 10% of the JIA population. Anti-citrullinated fibrinogen antibodies correlated significantly with IgG and IgA anti-CCP antibodies and IgA and IgM RF (p<0.05). Significantly elevated levels of IgG, IgM, and IgA anti-CCP antibodies, and IgA and IgM RF were noted in JIA patients who were also positive for anti-citrullinated fibrinogen antibodies. IgM RF and anti-citrullinated fibrinogen antibodies demonstrated the highest sensitivity for IgM RF-positive polyarthritis (100% and 81.3%, respectively) and JIA overall (43.4% and 32.3% respectively). IgG and IgA anti-CCP antibodies and anti-citrullinated fibrinogen antibodies exhibited the highest specificity for JIA (96.6%, 86.2%, and 86.2%, respectively).

## Conclusion

Of the antibodies measured in this study, anti-citrullinated fibrinogen antibodies showed the strongest association with JIA when compared to healthy and SLE control groups. Additionally, anti-citrullinated fibrinogen antibodies demonstrated high sensitivity and specificity for IgM RF-positive polyarthritis patients, along with IgG anti-CCP antibodies and IgM RF. Our data would suggest that measuring anti-citrullinated fibrinogen antibodies, in addition to anti-CCP antibody isotypes and IgM RF, may be beneficial in identifying patients that will develop more aggressive disease. For the first time, we have identified fibrinogen as a potential target for citrullination in JIA, particularly in patients with IgM RF-positive polyarticular JIA.

## Disclosure

Brooke E. Gilliam: None; Melinda R. Reed: None; Anil K. Chauhan: None; Amanda Dehlendorf: None; Peri H. Pepmueller: None; Terry L. Moore: None.

